# Impact of SWCNT-conjugated senna leaf extract on breast cancer cells: A potential apoptotic therapeutic strategy

**DOI:** 10.1515/biol-2022-0994

**Published:** 2024-12-31

**Authors:** Sabreen Mohammed Behairy, Saleh Mohammed Al-Maaqar, Majed Ahmed Al-Shaeri

**Affiliations:** Department of Biological Sciences, Faculty of Science, King Abdulaziz University, Jeddah, Saudi Arabia; Environmental Protection & Sustainability Research Group, Faculty of Science, King Abdulaziz University, Jeddah, Saudi Arabia; Biology, Faculty of Education, Al-Baydha University, Al-Baydha, Yemen

**Keywords:** breast cancer, drug delivery, senna leaf extracts, carbon nanotubes, DNA damage, cell viability

## Abstract

Breast cancer (BC) has a prevalence rate of 21.8% among Saudi women and ranks as the third leading cause of death in Western nations. Nanotechnology offers innovative methods for targeted BC therapy, and this study explores the use of single-walled carbon nanotubes (SWCNTs) for delivering the senna leaf extract. The study evaluated the effects of increasing dosages of senna leaf extract conjugated to SWCNTs on MCF-7 cells. Cell viability was assessed using the MTT assay, while Giemsa staining revealed morphological changes. Additionally, the comet assay and agarose gel electrophoresis were employed to evaluate the pro-apoptotic potential. The potential of mitochondrial membrane and the production of reactive oxygen species (ROS) were investigated using the JC-1 dye. The results indicated that treated cells exhibited apoptotic characteristics, including elevated ROS levels and decreased mitochondrial membrane potential. In summary, the application of nanotechnology to deliver the senna leaf extract shows promise as a herbal treatment for BC, suggesting a potential breakthrough in combating this widespread and deadly disease.

## 1 Introduction

Herbal remedies have a long history that dates back to ancient civilizations all over the world. Significant advancements in phytochemistry and phytopharmacology have led to a better understanding of the molecular makeup and biological characteristics of many medicinal plant products [1]. Active compounds in therapeutic plant species, such as flavonoids, tannins, and terpenoids, are crucial for their effectiveness; however, their high water solubility, large molecular size, and inability to pass through lipid cell membranes often result in low bioavailability and absorption challenges [2,3]. Given these difficulties, the combination of herbal medicine and nanotechnology has generated a lot of attention and discussion. The reason for this interest is that plant extracts may become more effective when formulated in nanostructured systems, which could result in lower dose requirements, fewer side effects, and better therapeutic outcomes [4,5,6]. By carefully directing them to the appropriate location of action, these nano-systems can provide a continuous and sufficient supply of active ingredients during the whole course of treatment [7,8]. Single-walled carbon nanotubes (SWCNTs) are essentially a single graphene sheet rolled into a seamless tube [9]. They have unique sp^2^-hybridized carbon surfaces and a sizable surface area, both of which are essential for efficient drug loading, including loading inside the tube [10,11,12]. The distinct physicochemical features of nanoparticles, specifically SWCNTs, have made them indispensable instruments in the field of nanomedicine. The remarkable mechanical strength, electrical conductivity, and surface area-to-volume ratio of SWCNTs make them suitable as carriers for targeted therapy and medication administration. SWCNTs are useful for selective photothermal ablation and cancer cell identification because of their significant near-infrared absorbance and Raman signals. Additionally, SWCNTs can be functionalized with a variety of molecules for improved therapeutic efficacy and targeted delivery, including aptamers and antibodies [13,14]. As a result, they have become extremely promising means of dispensing chemotherapies and cancer diagnostics. They have exceptional cell membrane penetrability, a high drug-carrying capacity, pH-dependent therapeutic release, prolonged circulation times, and intrinsic features like fluorescence, photothermal response, photoacoustic capabilities, and Raman properties [15,16,17,18]. These are the main causes of their exceptional qualities.

Senna, a genus classified within the Fabaceae family, Caesalpinioideae subfamily, and Cassieae tribe, specifically in the Aphyllae series, encompasses approximately 350 species of woody shrubs and subshrubs [19,20]. It was distinguished from the broader *Cassia* genus through the recognition of three distinct genera: senna, *Cassia* L. (s.s) and *Chamaecrista* Moench [21,22]. Many natural plant parts, including leaves, pods, roots, and fruits, have beneficial pharmacological properties that can be used to treat a variety of ailments. A wide range of actions, including anti-infectious, antioxidant, anticryptococcus, antitumor, antimutagenic, antiplasmodial, anti-inflammatory, anti-cancer, anti-diabetic, wound healing, and antihelminthic properties, are included in the studied pharmacological potentials of senna plants [23,24]. According to several studies, the phenolic and flavonoid contents of senna plants are what give them their antidiabetic effects [25]. These anti-diabetic effects work via processes that involve a decrease in glucose absorption and downregulation of several adipokines [26]. Saudi Arabia has easy access to the senna plant, a tiny shrub from the Caesalpiniaceae family. For the treatment of constipation, senna leaves are frequently used in over-the-counter drugs and herbal supplements [27]. Moreover, its leaves and fruit are used to make medicine for laxative disorders. It is useful in habitual constipation. According to pharmacological research, sennosides A and B are responsible for all of the action in senna leaves and pods. Senna leaves do, however, contain the glycosides sennosides A, B, C, and D. The leaves and pods of the senna plant have already yielded the two naphthalene glycosides [28].

To effectively target the senna (*Cassia angustifolia*) leaf extract for the treatment of breast cancer (BC), this study aimed to develop highly efficient drug delivery systems based on SWCNTs. The crude extract of senna leaves was prepared and subsequently conjugated to the SWCNTs. Additionally, the cell viability assay, cell migration assay, and comet assay were performed to validate the effectiveness of the formulated delivery system. Cellular morphological changes were evaluated for both SWCNTs alone and the combination of senna leaf extract with SWCNTs. Furthermore, the generation of reactive oxygen species (ROS) and the assessment of mitochondrial membrane potential provided additional insights into the therapeutic potential of the formulated drug delivery system for BC treatment.

## 2 Materials and methods

### 2.1 Preparation of crude extracts

Fresh senna leaves were obtained from a local market in Jeddah, Saudi Arabia, and their authenticity was confirmed by taxonomic specialists at King Abdulaziz University (voucher number: *Anethum graveolens* L. dill #AG17600). The leaves were soaked in 75% ethanol for 7 days at room temperature to extract their chemical components. The resulting macerate was filtered using Whatman filter paper and evaporated with ethanol in a rotary evaporator. An equal volume of *n*-hexane was added to the residue, which was stirred for 3–4 h and then separated using a separating funnel. The aqueous portion was retained for further processing with ethyl acetate, following a similar stirring and separation process. The ethyl acetate fraction was collected, evaporated, and stored as a stock solution at 4°C. A fresh working standard of the ethyl acetate fraction was prepared by diluting the stock solution in 100% DMSO (dimethyl sulfoxide).

### 2.2 Synthesis and characterization of SWCNTs

SWCNTs were purchased from a commercial supplier as a stock solution in distilled water at a concentration of 1 mg per liter (diameter 1.1 nm × length 0.5–100 μm). 0.02% Suwannee River Natural Organic Matter was used as a dispersant to help in dispersion. Before being utilized, the SWCNT stock underwent a 2 h ultrasonic dispersion procedure utilizing the Decon FS300 Frequency Sweep apparatus. This made the solution acceptable for a variety of applications by ensuring an extensive and uniform dispersion of SWCNTs in solution.

### 2.3 SWCNT preparation and drug conjugation

SWCNTs were briefly sonicated with polyvinylpyrrolidone polymer to create a uniform and stable dispersion of SWCNTs in water. The SWCNTs were then suspended in distilled water, washed repeatedly with deionized water until the pH reached 6–7, neutralized, and dried. About 1.2 mg of SWCNTs was combined with 1 mL of phosphate-buffered saline (PBS). To enable active targeting of BC cells, the SWCNTs were conjugated with 6 mg of 1-ethyl-3-[3-dimethylaminopropyl]carbodiimide (EDC) and 6 mg of *N*-hydroxysulfosuccinimide (NHS) using the active ester technique. The mixture was rocked for 30 min at room temperature, sonicated for 30 min in a water bath, and incubated for another 24 h. The resulting mixture was filtered using a vacuum pump and 0.45 μm polytetrafluoroethylene membrane filters. Finally, physicochemical interactions with the surfaces of SWCNTs enabled the binding of excised senna leaves.

### 2.4 Cell culturing

In the process of subculturing, human Michigan Cancer Foundation-7 (MCF7) cells were obtained from King Abdulaziz University, specifically from the King Fahd Center for Medical Research in Jeddah, Saudi Arabia. The MCF7 human BC cells were cultivated according to the manufacturer’s instructions in a controlled tissue culture environment using Dulbecco’s modified Eagle’s medium (DMEM, Gibco), supplemented with 10% fetal bovine serum and 1% penicillin–streptomycin antibiotics. The culture was maintained at a constant temperature of 37°C and a 5% CO_2_ atmosphere. Following the 2012 protocol from the American Type Culture Collection, the cells were sub-cultured every 3–4 days. All experiments were conducted under these optimized conditions to ensure the viability and growth of MCF7 cells.

### 2.5 Cell viability assessment

The MTT assay (3-(4,5-dimethylthiazol-2-yl)-2,5-diphenyltetrazolium bromide) from CELL BIOLABS, INC. was used to assess the viability of MCF7 cell population. Initially, seeding of MCF7 cells onto 96-well plates was done at a density of 10 × 10^3^ cells per well, and the cells were allowed to grow overnight. The MC7F7 cells were then incubated for 24 h while being exposed to the extract at various doses. After the initial incubation, MTT reagent was added to each well, and the plates underwent an additional 4 h of incubation. Following the 4 h incubation, 100 μL of DMSO (crystal dissolving solution) was added to each well, and the plates were allowed to continue to incubate at room temperature for the remaining night. By measuring the converted dye’s absorbance at a wavelength of 570 nm and correlating the level of enhanced absorbance with cell viability, it was possible to get important insights into how the extract affected the proliferation and survival of MCF7 cells [29,30,31].

### 2.6 Cell migration assay

**Figure 1:**
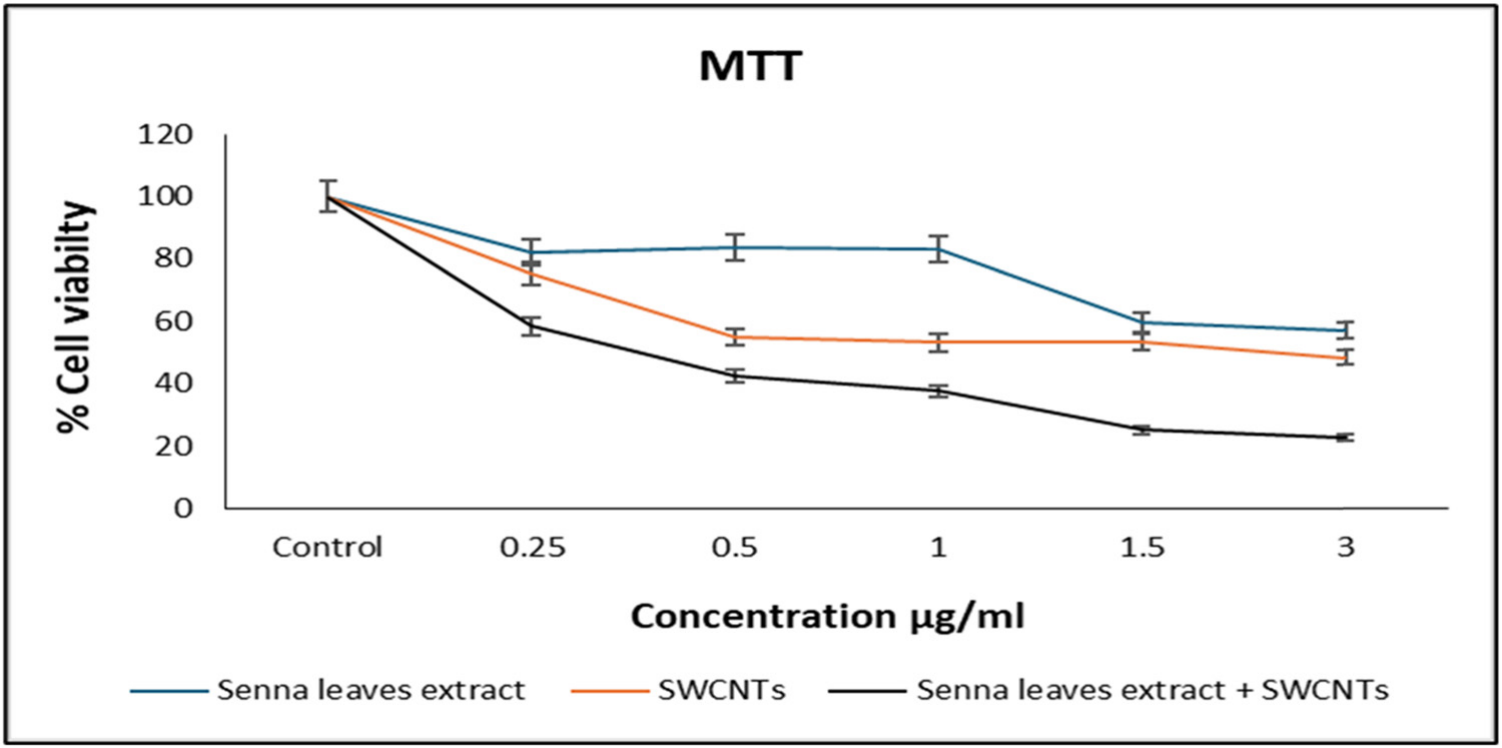
Effect of senna leaf extract alone, SWCNTs alone, and senna leaf extract + SWCNTs on the viability of MCF7 cells (10 × 10^3^ cells/well) following 24 h of treatment. Decrease of the viability in a dose-dependent manner is shown.

The exact steps taken in the wound healing assay were as follows: cells were initially plated in a 24-well plate at a density of 4 × 10^3^ cells per well. Over the course of a 24 h incubation period, these cells were allowed to develop and form a monolayer. A pipette tip was used to make a control “wound.” By gently scraping the cell monolayer, a space or “wound” was created in the cell culture, which was done carefully. The culture medium was then swapped out for new medium after the wound had been created. The cells were then treated with SWCNTs in various doses together with the senna leaf extract. These concentrations ranged from 0.25 to 3 μL/mL, as shown in Figure 1. The process of cell migration was monitored and documented by capturing images of the wound area at specific time points, with a particular focus on imaging after a 24-h incubation period. This experiment gave researchers a way to observe and measure how cells moved in response to the treatments they were given. The experiment shed light on their possible impact on cell mobility and the wound healing process by investigating the effects of various dosages of SWCNTs in combination with the senna leaf extract. This experiment gave researchers a way to observe and measure how cells moved in response to the treatments they were given. It also shed light on their possible impact on cell mobility and the wound healing process by investigating the effects of various dosages of SWCNTs in combination with the senna leaf extract.

### 2.7 Comet assay

Single-cell gel electrophoresis, also referred to as the comet assay, was used to measure the degree of deoxyribonucleic acid (DNA) damage. A thorough explanation of the process is provided below: different concentrations of SWCNTs mixed with the senna leaf extract (0.25, 0.5, 1.0, 1.5, and 3 μL/mL), as well as an additional 0.5 μL of SWCNTs, were used to treat the cells under varied circumstances. Cells were kept in a full medium throughout the course of the 24-h treatment. Cells were carefully removed following the treatment period and suspended in cold PBS. The comet assay’s subsequent procedures were completed in carefully regulated subdued light settings to reduce any potential light-induced DNA damage as described earlier [32]. Slides of a comet were specifically prepared for analysis. These slides underwent electrophoresis while containing the suspended cells. Using a fluorescence microscope, the cell nuclei were observed and photographed at 10× and 20× magnifications.

### 2.8 Assessment of cell morphological changes

To investigate the nuclear morphological changes induced by apoptosis, Giemsa staining was employed. Cells were initially seeded in 24-well plates at a density of 4 × 10^3^ cells per well. After then, the culture media was changed, and different doses of a mixture of senna leaf extract and SWCNTs were administered to the cells. The cells were thoroughly rinsed with 1× cold PBS after the 24-h treatment period. In order to maintain the fixed-cell samples, a 10% formalin-based buffer was used. The fixed cells were stained with Giemsa at a concentration of 0.25%. The Giemsa-stained cell samples were then examined with an inverted microscope at 20× magnification. By employing Giemsa staining and microscopy analysis, the morphological features associated with apoptosis were observed and documented.

### 2.9 Determination of ROS production

To evaluate the production of ROS, 2′,7′-dichlorofluorescein diacetate (DCFH-DA; Cayman Chemicals, USA) was used. In 96-well plates, MCF7 cells were initially grown. The cells were subsequently treated for 24 h with various concentrations of a mixture of SWCNTs and senna leaf extract. The cells were tagged with DCFH-DA for 30 min following the treatment period. The cells were thoroughly rinsed with PBS after the labeling stage. With excitation at 485/20 nm and emission at 528/20 nm, a microplate reader [33] was used to measure the fluorescence intensity. This process made it possible to measure the ROS production in the treated MCF7 cells. The quantities of ROS generated in response to various concentrations of SWCNTs and senna leaf extract combination were evaluated by DCFH-DA and fluorescence assays.

### 2.10 Analysis of MMP

MCF7 human cancer cells were grown for mitochondrial analysis. After a 15-min initial incubation period, mitochondria were tagged by incubating them with JC-1 (12.5 M) for 20 min. The culture medium, DMEM without phenol red and bicarbonate, was then supplemented with the labeled mitochondria. Following a protocol outlined by Bernas and Dobrucki [34], the culture medium containing the mitochondrial label was removed after the incubation period, the cells were washed, and new medium was added.

### 2.11 Statistical analyses

The data from our experiments are presented as mean values with accompanying standard deviations (mean ± SD). To evaluate the variations between the different samples, statistical analyses were performed. One-way analysis of variance was used for these studies. A significance level of *P* ≤ 0.05 was chosen to determine the statistical significance.

## 3 Results

### 3.1 SWCNTs combined with senna leaf extract inhibit cell vitality and cell migration in MCF7 cells

MCF7 cells were exposed to various concentrations of senna leaf extract (0.25, 0.5, 1.0, 1.5, and 3 μg/mL) + 0.5 μg SWCNTs to study their effect on cell proliferation (Table 1). The MTT assay revealed the cell proliferation inhibition. Treatment of MCF7 cells with SWCNTs and senna leaf extract results in a concentration- and time-dependent positive decline in viability, as mentioned in Figure 1. It is usual practice to measure a drug’s potency using its half-maximum inhibitory concentration (IC_50_) value; the lower the IC_50_ number, the more potent the drug is.

**Table 1:** Determination of cytotoxicity of SWCNTs + senna, senna alone, and SWCNTs alone to MCF7 cells by the MTT assay

Concentrations						
**Treatment 1**	Control	0.25	0.5	1	1.5	3
SWCNTs alone	100 ± 0.17	99.3 ± 0.39	99.5 ± 0.14	73.8 ± 0.290	53.7 ± 0.1	47.2 ± 0.05
**Treatment 2**	Control	0.25	0.5	1	1.5	3
Senna extract alone	100 ± 0.3	83.5 ± 0.3	84.2 ± 0.25	70.5 ± 0.12	60.4 ± 0.45	55.8 ± 0.31
**Treatment 3**	Control	0.25 + 0.5	0.5 + 0.5	1 + 0.5	1.5 + 0.5	3 + 0.5
Senna extract + SWCNTs	100 ± 0.2	58.03 ± 0.1	48.2 ± 0.04	34.5 ± 0.14	29.7 ± 0.2	22.2 ± 0.12

### 3.2 Synergistic effects of senna plant and SWCNTs on cell migration in MCF7 cells

**Figure 2:**
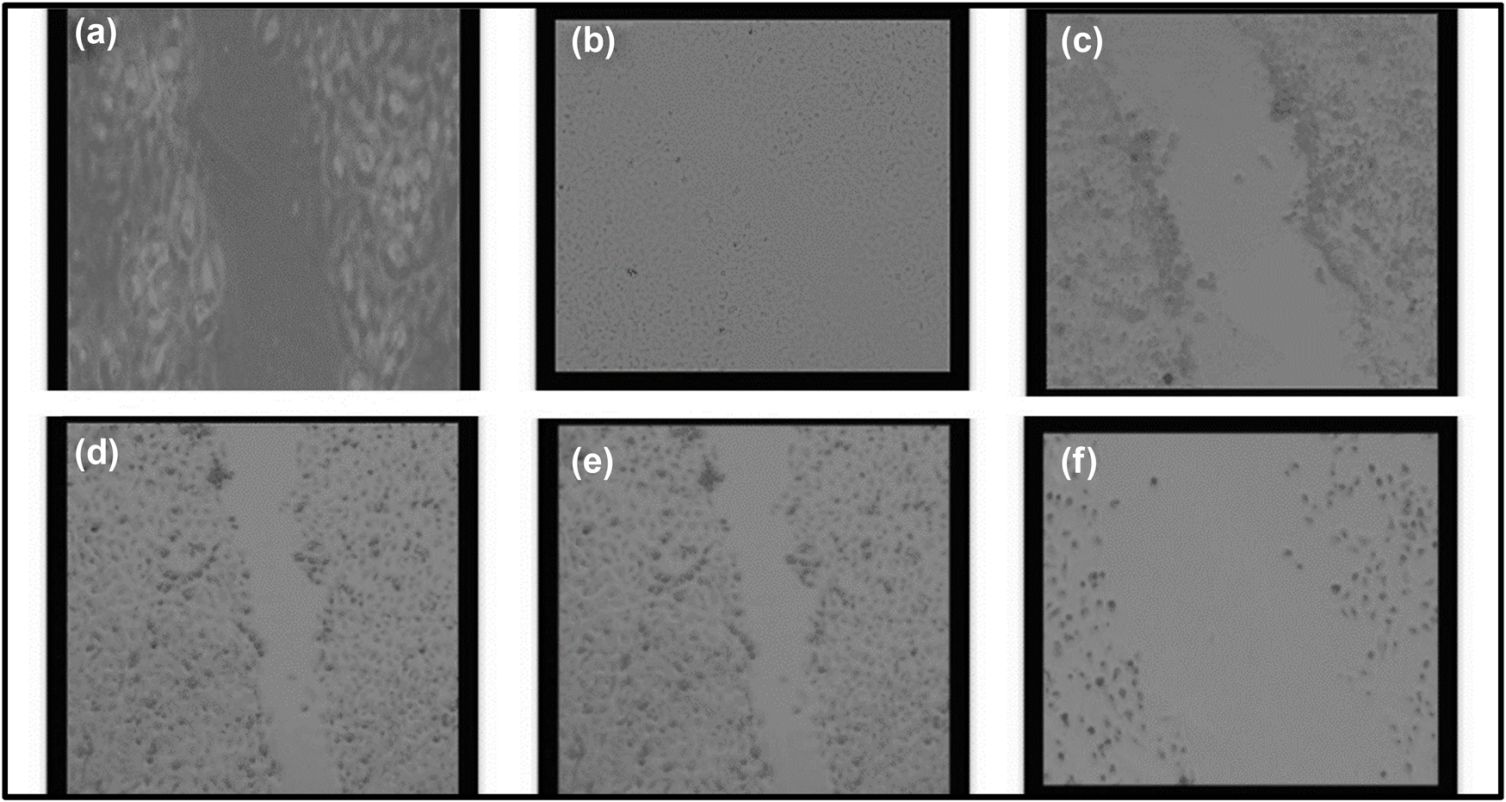
Cell migration assay for the effect of senna leaf extract + SWCNTs on MCF7 cells. Photomicrographs showing different doses (0.25, 0.5, 1.0, 1.5, and 3 μg/mL) induced cell migration in MCF7 cells after 24 h. Cells were observed under a light microscope at 10× magnification. (a) Control, (b) 0.25 µg/mL senna extract + 0.5 µg/mL SWCNTs, (c) 0.5 µg/mL senna extract + 0.5 µg/mL SWCNTs, (d) 1 µg/mL senna extract + 0.5 µg/mL SWCNTs, (e) 1.5 µg/mL senna extract + 0.5 µg/mL SWCNTs, and (f) 3 µg/mL senna extract + 0.5 µg/mL SWCNTs.

The wound healing assay was employed to monitor cellular responses. This methodology provides us with the capability to observe and assess cell behaviors and reactions. The wound was made by using a pipette tip in a cell monolayer. The wound healing (cell migration) was recorded by taking pictures. Figure 2 shows that senna plant in combination with 0.5 µg SWCNTs induced cell migration in MCF7 cells. Control showed that the cells started to migrate and the wound disappeared, while in treated cells the wound remained large and apparent in the case of treatment with different doses (0.25, 0.5, 1.0, 1.5, and 3 μg/mL).

### 3.3 SWCNTs and senna together change the morphology of MCF7 cells

**Figure 3:**
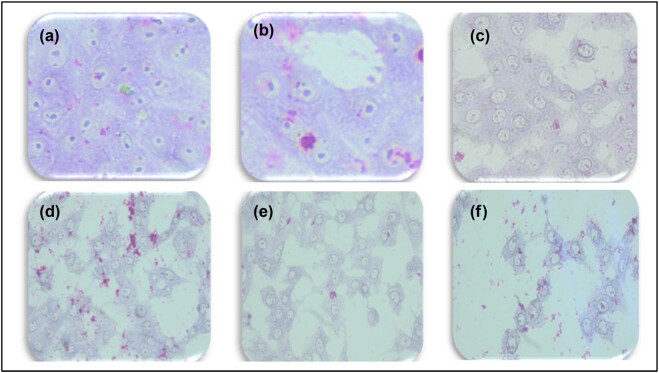
Morphological changes evident of apoptosis in MCF7 cells detected using bright-field microscopy imaging in fixed and Giemsa-stained cell samples with 20× magnification. Cells were treated with indicated concentrations of senna leaf extract (0.25, 0.5, 1.0, 1.5, and 3 μg/mL) + SWCNTs 0.5 μL. (a) Control, (b) 0.25 µg/mL senna extract + 0.5 µg/mL SWCNTs, (c) 0.5 µg/mL senna extract + 0.5 µg/mL SWCNTs, (d) 1 µg/mL senna extract + 0.5 µg/mL SWCNTs, (e) 1.5 µg/mL senna extract + 0.5 µg/mL SWCNTs, and (f) 3 µg/mL senna extract + 0.5 µg/mL SWCNTs.

**Figure 4:**
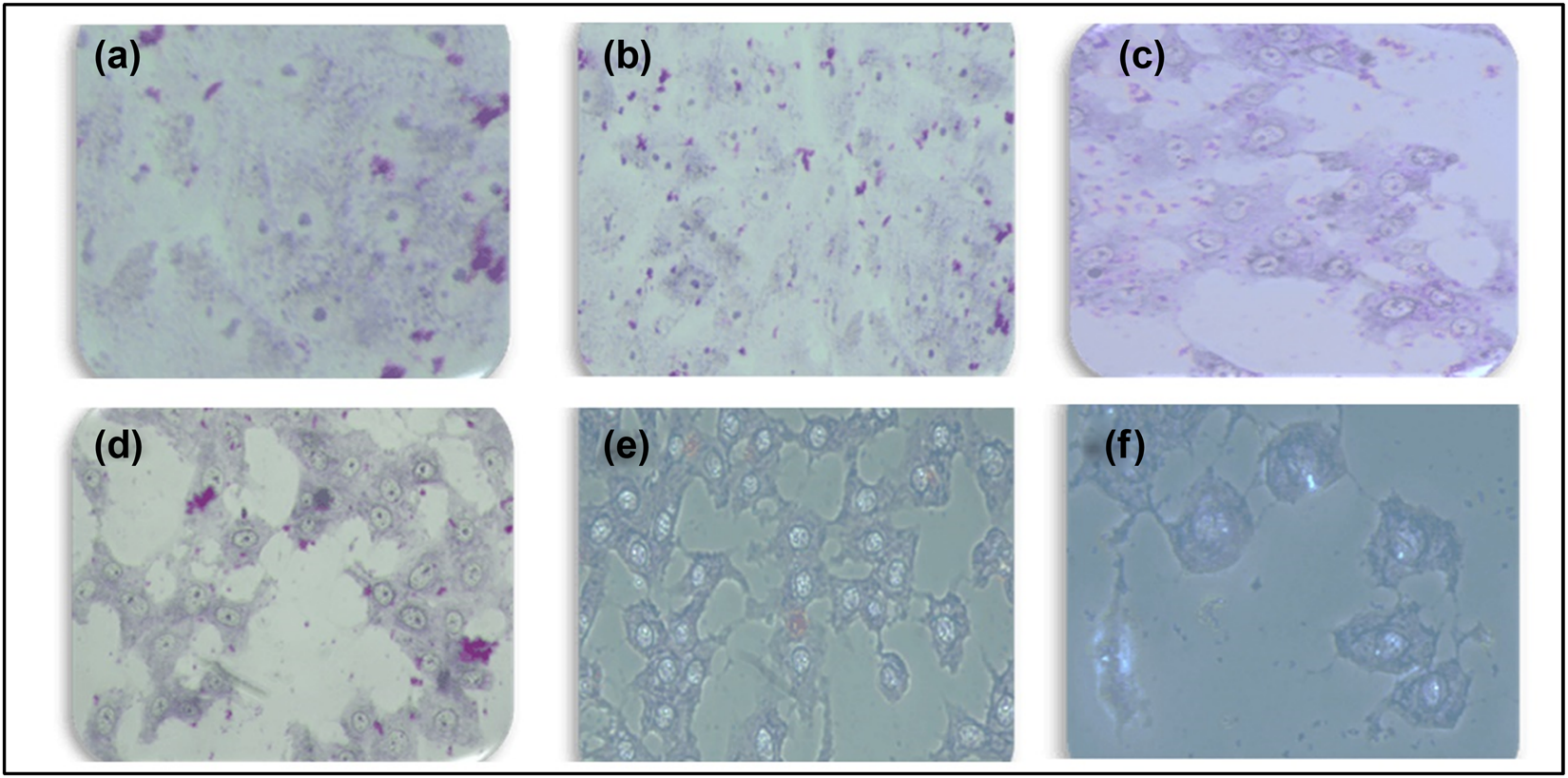
Morphological changes indicative of apoptosis in MCF7 cells were visualized through bright-field microscopy imaging of fixed and Giemsa-stained cell samples, utilizing a 20× magnification. The cells were subjected to treatment with specified concentrations of senna leaf extract only (0.25, 0.5, 1.0, 1.5, and 3 μg/mL). (a) Control, (b) 0.25 µg/mL senna extract + 0.5 µg/mL SWCNTs, (c) 0.5 µg/mL senna extract + 0.5 µg/mL SWCNTs, (d) 1 µg/mL senna extract + 0.5 µg/mL SWCNTs, (e) 1.5 µg/mL senna extract + 0.5 µg/mL SWCNTs, and (f) 3 µg/mL senna extract + 0.5 µg/mL SWCNTs.

**Figure 5:**
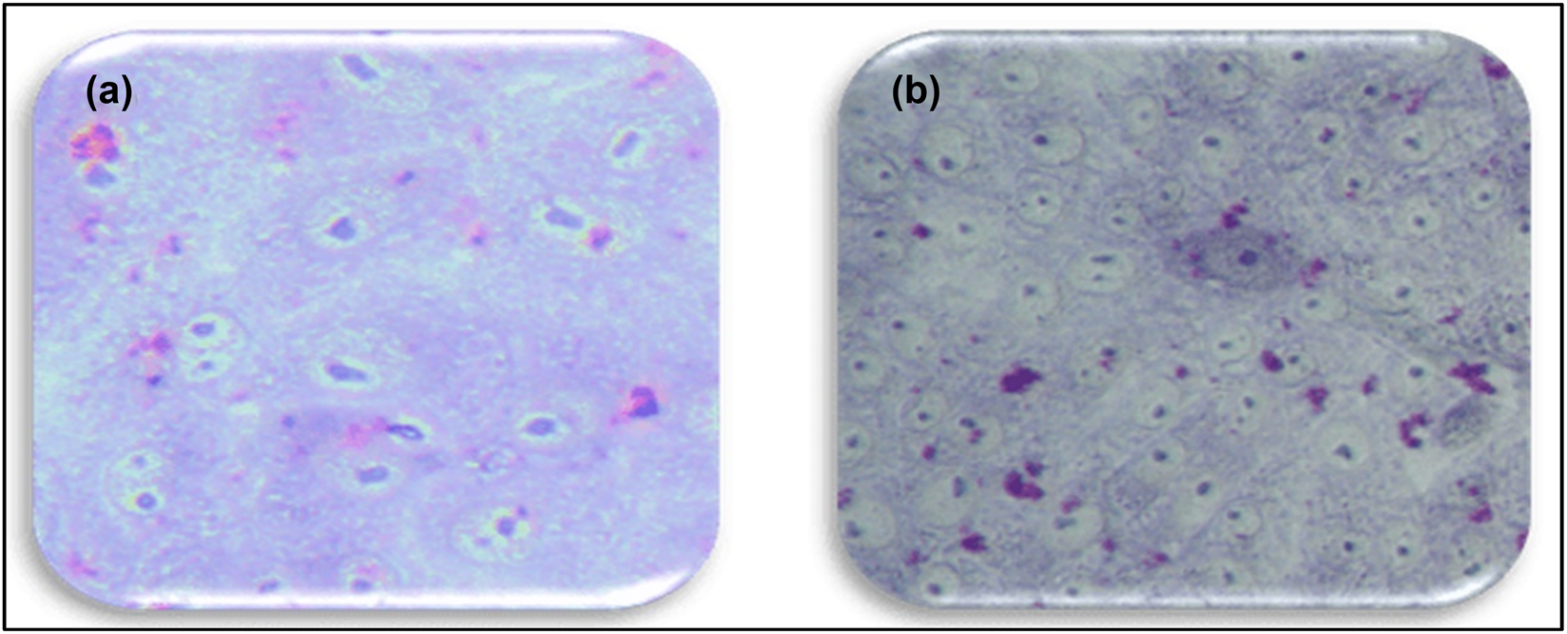
Demonstration of the absence of morphological alterations in MCF7 cells following treatment with SWCNTs alone, as observed through bright-field microscopy imaging of fixed and Giemsa-stained cell samples at a 20× magnification.

Giemsa staining was used to examine the effects of senna leaf extract paired with SWCNTs on cell morphology. Figures 3–5 show that untreated MCF7 cells grew quickly and confluently into a monolayer with uniformly stained nuclei. Cells treated with senna leaf extract and SWCNTs, on the other hand, showed unique morphological signs of apoptosis. With increasing senna leaf extract and SWCNT concentrations, these characteristics grew more pronounced. Cell shrinkage, the appearance of condensed chromatin masses, nuclear fragmentation, and the development of apoptotic bodies were all apoptotic features.

### 3.4 DNA damage induced by senna and SWCNTs without oligo-nucleosomal degradation

**Figure 6:**
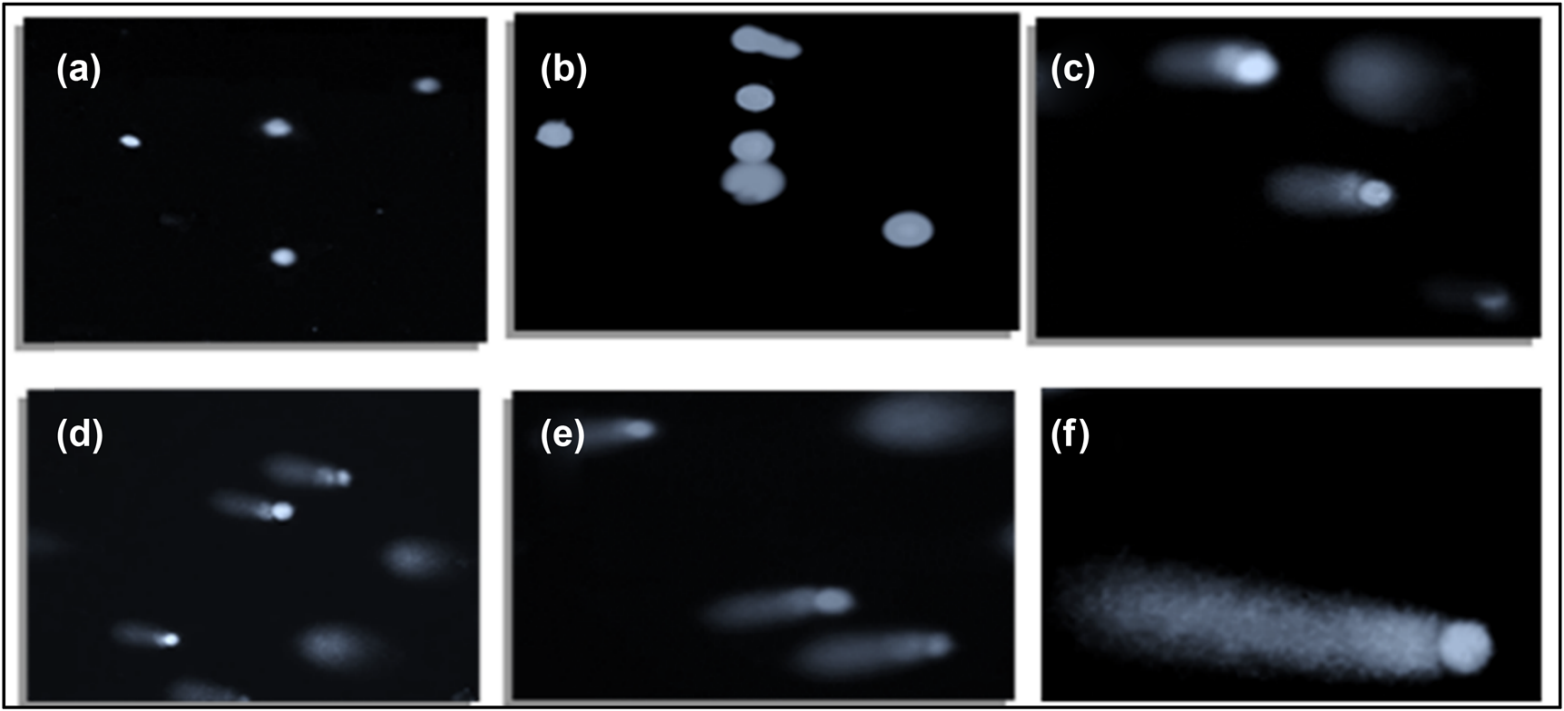
Apoptotic comet formation induced by senna leaf extract + SWCNTs. (a) Control, (b) 0.25 µL + 5 µL, (c) 0.5 µL + 0.5 µL, (d) 1.0 µL + 0.5 µL, (e) 1.5 µL + 0.5 µL, and (f) 3 µL + 0.5 µL treatments on MCF7 cells. The images clearly illustrate the presence of comet formations, a characteristic hallmark of apoptosis, at a 10× magnification.

The comet assay was carried out, as shown in Figure 6, to evaluate the possible induction of DNA damage by SWCNTs paired with senna leaves in MCF7 cells. With this test, DNA double-strand breaks are particularly found. DNA and nuclei in untreated cells had a spherical shape, and no tail could be seen. However, in the beginning, the movement of DNA out of the nucleus was seen in cells treated with the lowest dose of SWCNTs (0.5 µL) and senna leaves (0.25 µL/mL), indicating DNA breakage, and the treated cells had incomplete comet tails. The nuclei of cells treated with the greatest concentration of SWCNTs (0.5 µL) conjugated to senna leaves (3.0 µL/mL) displayed the appearance of a bright comet with a tail, indicating the loss of DNA structure. It is important to remember that the comet assay should be carried out meticulously to prevent false-positive results that can result from DNA damage linked to apoptosis rather than genotoxicity [34]. This leads us to the conclusion that the observed DNA damage occurs during apoptosis using a non-classical DNA fragmentation pathway.

### 3.5 Senna plant and SWCNTs elicit ROS accumulation and influence mitochondrial membranes in MCF7 cells

**Figure 7:**
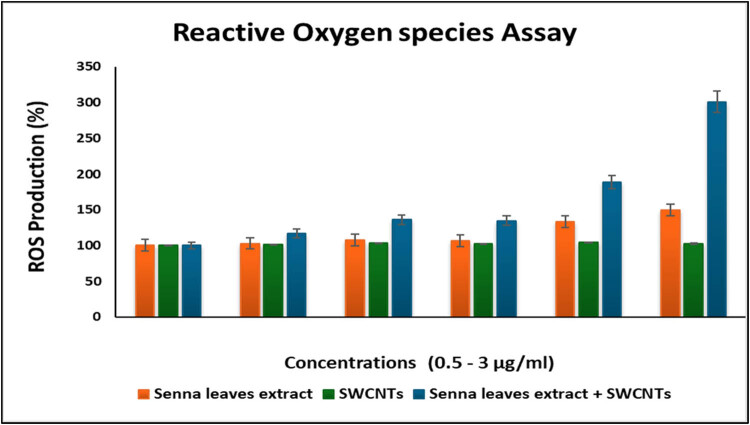
Combination of senna leaf extract and SWCNTs triggers a dose-dependent increase in the production of ROS in MCF7 cells, with statistical significance (*P* < 0.05) observed when compared to the control.

**Figure 8:**
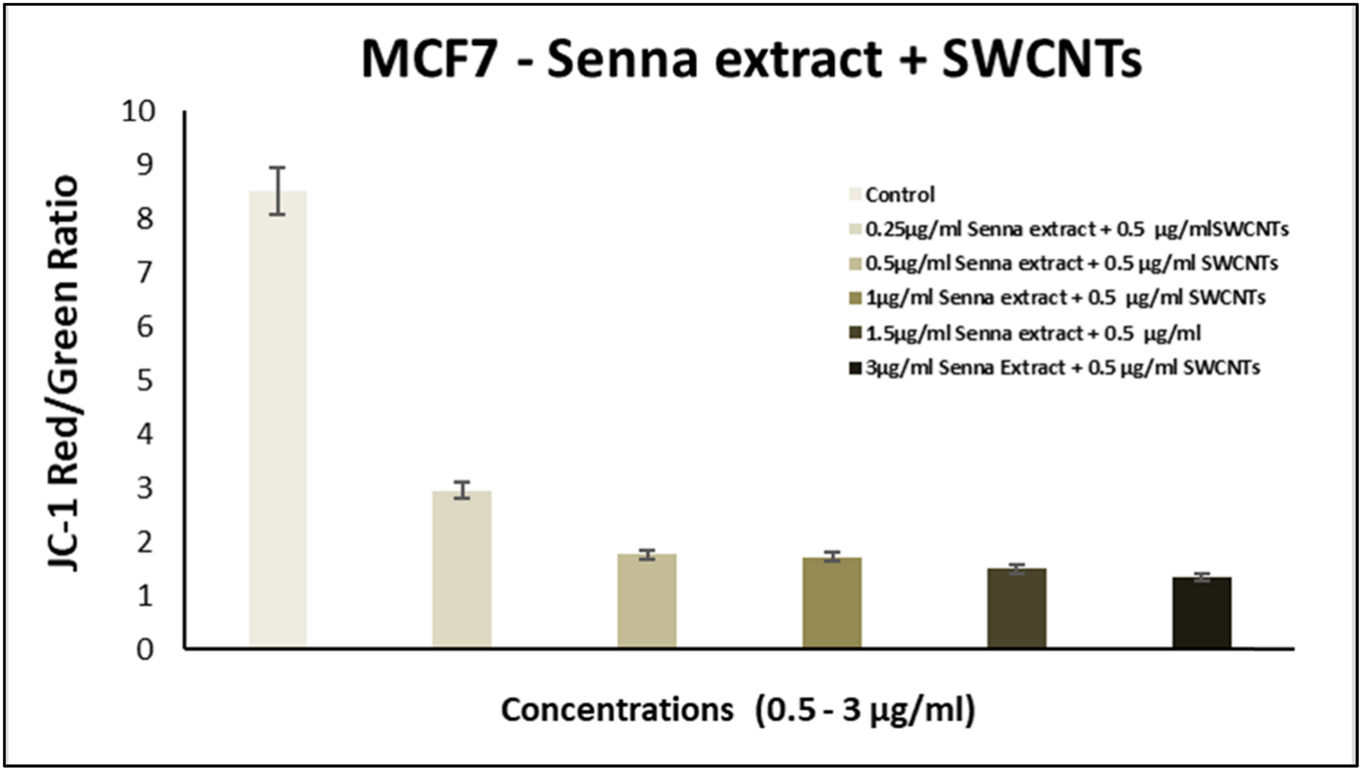
Impact of exposing MCF-7 cells to escalating concentrations of senna leaf extract + SWCNTs for a 24 h period on mitochondrial membrane potential was assessed using the JC-1 fluorescence assay. Each bar in the graph signifies the mean value, and the error bars depict the standard deviation (SD). Significance levels are denoted as follows: *P ≤ 0.05 = significant, **P < 0.01 = highly significant.

**Figure 9:**
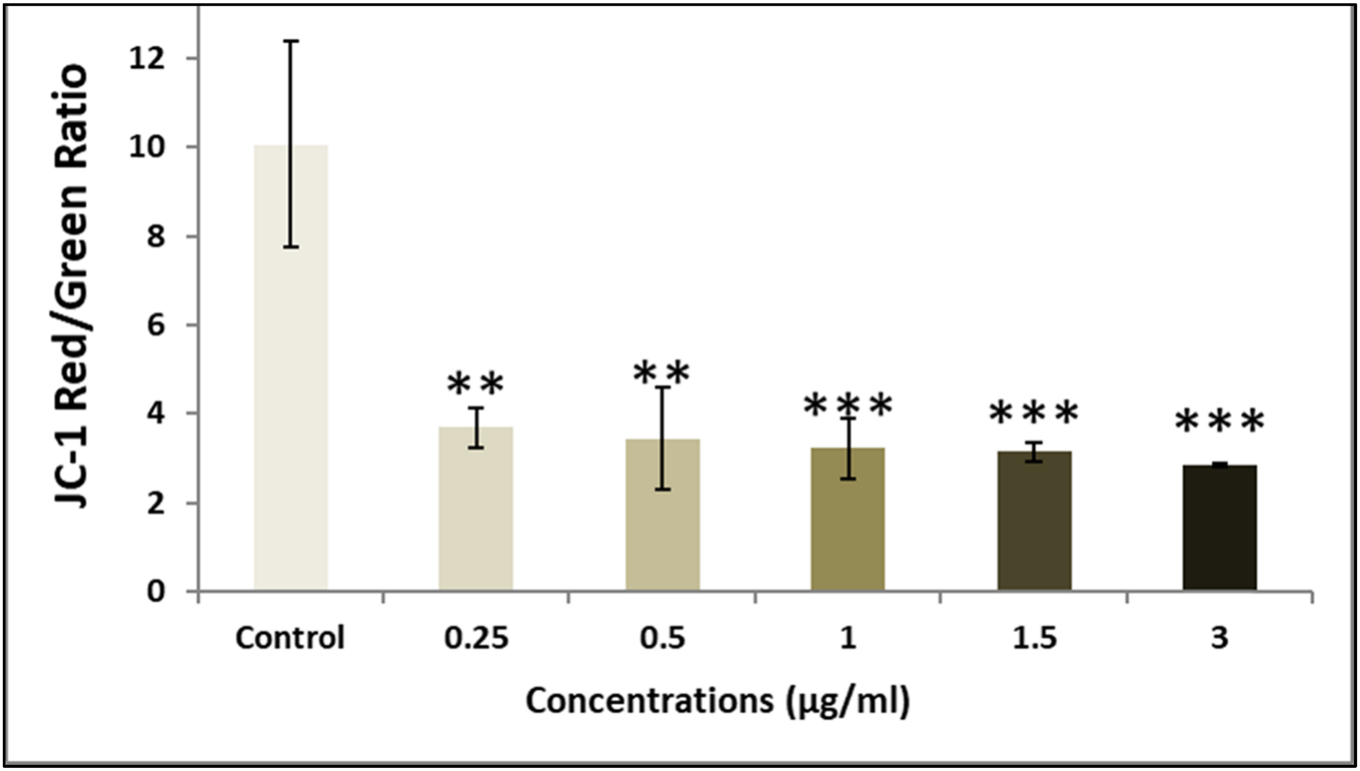
Influence of subjecting MCF-7 cells to escalating concentrations of senna extract alone for a duration of 24 h on mitochondrial membrane potential was evaluated using the JC-1 fluorescence assay. Each bar in the graph corresponds to the mean value, while the error bars represent the standard deviation (SD). Significance levels are indicated as follows: *P ≤ 0.05 = statistically significant, **P < 0.01 = highly statistically significant.

**Figure 10:**
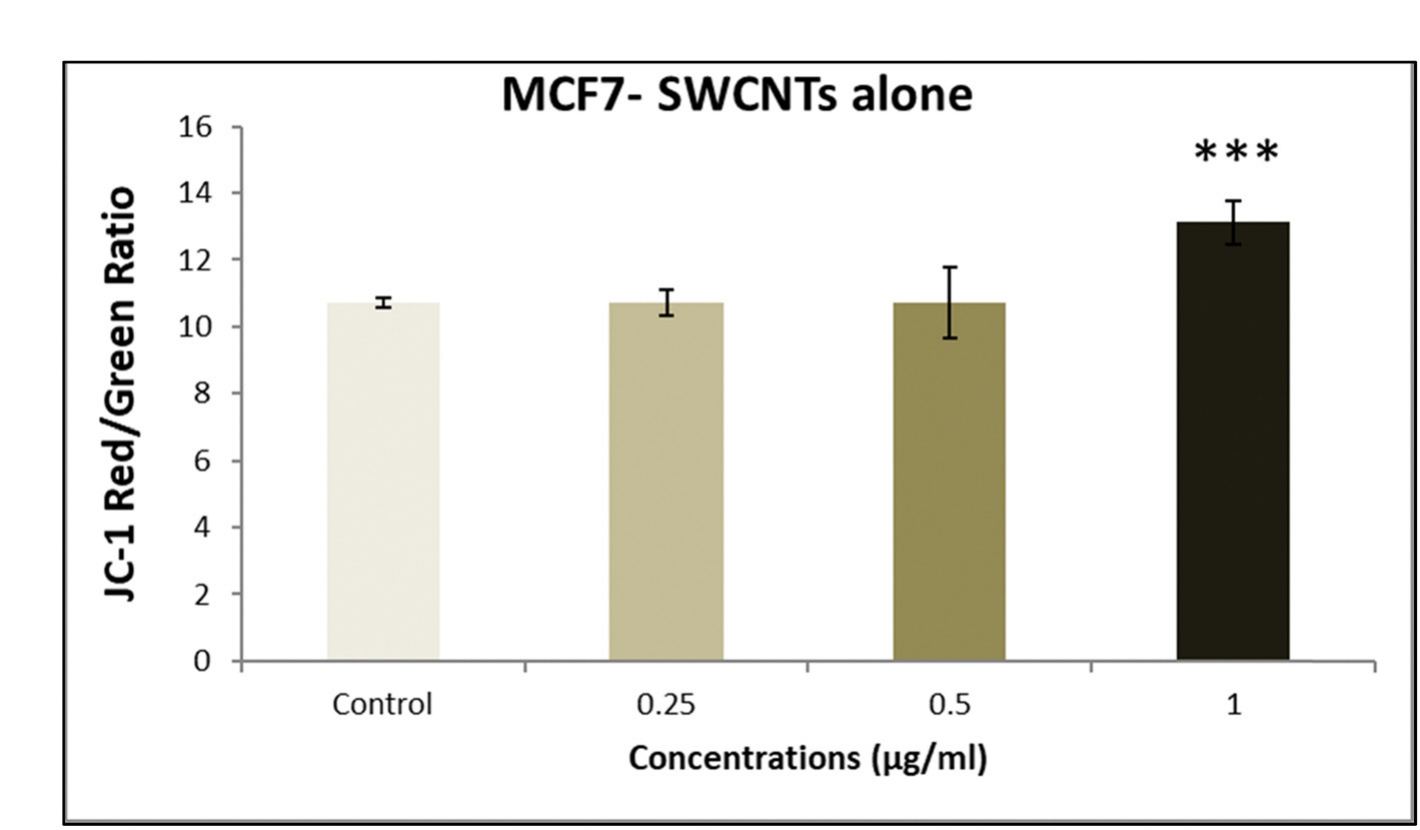
Impact of exposing MCF-7 cells to escalating concentrations of SWCNTs in isolation for a 24 h duration on mitochondrial membrane potential, as determined through the JC-1 fluorescence assay.

We examined intracellular ROS levels during the treatment to investigate whether the combination of senna extract and SWCNTs induces apoptosis through ROS generation. Figure 7 illustrates the dose-dependent elevation of ROS production induced by increasing concentrations of senna and SWCNTs (Table 2). Elevated ROS levels are recognized for their role in outer mitochondrial membrane permeabilization (MMP), leading to the loss of mitochondrial membrane potential and the release of pro-apoptotic proteins into the cytoplasm [32]. By observing the changes in the red/green ratio of JC-1 staining, the damage to the mitochondria was further demonstrated. Using a fluorescence microplate reader, the intensities of the colors red and green were measured. According to Figures 8–10, when compared to untreated cells, the senna extract + SWCNT treatment dose-dependently decreased the red/green ratio of JC-1 staining in MCF7 cells. This decrease in the red/green ratio suggests a problem with mitochondrial activity, pointing to a link between ROS buildup and mitochondrial malfunction in the observed cellular response.

**Table 2:** ROS levels expressed in terms of percent of control and presented as mean ± SD and *P* ≤ 0.05

Concentrations						
**Treatment 1**	Control	0.25	0.5	1	1.5	3
SWCNTs alone	0.53 ± 0.12	0.51 ± 0.05	0.53 ± 0.14	0.81 ± 0.05	0.96 ± 0.71	1.3 ± 0.21
**Treatment 2**	Control	0.25	0.5	1	1.5	3
Senna extract alone	0.51 ± 0.1	0.82 ± 0.5	0.85 ± 0.02	1.2 ± 0.01	1.21 ± 0.32	1.31 ± 0.5
**Treatment 3**	Control	0.25 + 0.5	0.5 + 0.5	1 + 0.5	1.5 + 0.5	3 + 0.5
Senna extract + SWCNTs	0.5 ± 0.07	1.2 ± 0.21	1.4 ± 0.03	1.46 ± 0.14	1.6 ± 0.6	2.4 ± 0.17

## 4 Discussion

BC continues to be one of the most common diseases in women worldwide, with high rates of morbidity and death. Novel approaches to therapy are desperately needed, especially if they can maximize the positive effects of current treatments while reducing their negative ones. Because of its special qualities – such as its large surface area, good conductivity, and capacity to be functionalized for targeted drug delivery – nanotechnology, and more specifically the utilization of SWCNTs, has become a promising new tool in the fight against cancer [35,36,37]. The use of nanoparticles in drug delivery systems has revolutionized the medical industry and offers a promising way to boost the therapeutic efficacy of natural products. With this breakthrough, the quest to optimize natural compounds’ potential for medical uses has made tremendous progress [38,39]. Therapeutic substances, such as naturally occurring chemicals produced from plants, can be efficiently transported to cancer cells via SWCNTs. The senna leaf extract, well known for its many bioactive constituents, has shown promise as an anticancer agent, even causing cancer cells to undergo apoptosis. It is conjugated with SWCNTs to improve its solubility, stability, cellular absorption, and bioavailability, which will ultimately result in more potent therapeutic effects [40]. Studies have demonstrated that SWCNTs may induce apoptosis via a variety of methods, including the production of ROS and interference with mitochondrial activity [35,41]. According to the research, SWCNTs, for example, can increase ROS levels in cancer cells, which triggers oxidative stress and the activation of apoptotic pathways like the caspase cascade. This is especially important in the case of BC, since pro-apoptotic and anti-apoptotic factors frequently have an unbalanced relationship that leads to tumor growth and treatment resistance. Moreover, SWCNTs’ capacity to selectively target particular cancer cell types expands their therapeutic range. Researchers have shown that functionalizing SWCNTs with targeting ligands allows for selective delivery to BC cells, reducing the possibility of off-target effects and enhancing therapy specificity [35,42,43]. The limits of conventional chemotherapy, which frequently damages healthy cells and causes serious adverse effects, must be overcome by using this focused technique. In this study, we set out to investigate the synergistic effects of mixing SWCNTs with extracts from senna leaves on the well-known MCF7 cell line, which represents human BC. The use of senna, a plant that is extensively cultivated throughout the world, in traditional herbal medicine is extensive. In addition to the treatment of constipation and piles, it has also been used to treat epilepsy, respiratory diseases, skin infections, migraine, and other conditions [44,45]. In order to explore senna’s potential as a powerful participant in the field of cancer therapy, our study gave special attention to its pharmacological importance. SWCNTs, recognized for their extraordinary properties and promising uses in medication delivery, were integrated with a lot of effort. SWCNTs’ innate anticancer capabilities persisted even after they were covalently linked to the senna leaf extract. This partnership reveals promising paths for cancer drug delivery and therapeutic treatments with a focus on apoptotic pathways, a crucial defense mechanism against cancer cells [40,46].

The main focus of our study was identifying the significant effects of this combo therapy. We found that the combination of SWCNTs and senna leaf extract inhibited BC (MCF7) cells in a dose- and time-dependent manner. The unique IC_50_ values obtained from cell viability studies highlighted the potential of this innovative strategy and are in perfect accordance with earlier study findings [32]. Our findings from the wound healing experiment provide important evidence for the existence of cell migration, a behavior closely related to cancer cell metastasis [47]. The outcomes confirmed the promise of this combination therapy in preventing the spread of cancer cells and were consistent with earlier research [48]. Staining assays were used to illuminate the mechanisms of cell death brought on by the therapy, revealing more information about the method of action. The potential of the combination therapy was confirmed by the presence of telltale signs of apoptosis in treated cells, including cell and nuclear shrinkage, chromatin condensation, and development of apoptotic bodies [44,49,50,51]. Our investigation into genetics went further, and we used the comet assay to measure DNA damage. Another aspect of this potential strategy was revealed when comet tails appeared in the treated cells, which offered a tangible proof to DNA damage and apoptosis [38,52,53,54]. Cell viability depends on healthy mitochondria, and damage to this organelle can trigger apoptotic processes. ROS and mitochondrial membrane potential (MMP) concentrations were measured in our investigation. Co-administration of the senna extract and SWCNTs provided insights into the apoptotic mechanisms at play, as evidenced by the dose-dependent elevation of ROS production and the perturbation of MMP.

## 5 Conclusions

In conclusion, our research demonstrates the enormous potential of synergistic medicines that combine the special qualities of SWCNTs with the potency of senna leaves. With a specific emphasis on apoptotic pathways and inhibition of cancer cell proliferation, this strategy offers prospective prospects for improved cancer therapy. The landscape of cancer treatment may change as a result of these discoveries, which open the door for additional strides in the field of cancer treatments.
